# Supplementary figures and images for: First detection of Koi herpesvirus disease (KHVD) in Garmian, Kurdistan region of Iraq: A clinical and molecular study
Source: PLoS One. 2024 May 31;19(5):e0303475. doi: 10.1371/journal.pone.0303475 (PMC11142564; doi:10.1371/journal.pone.0303475)

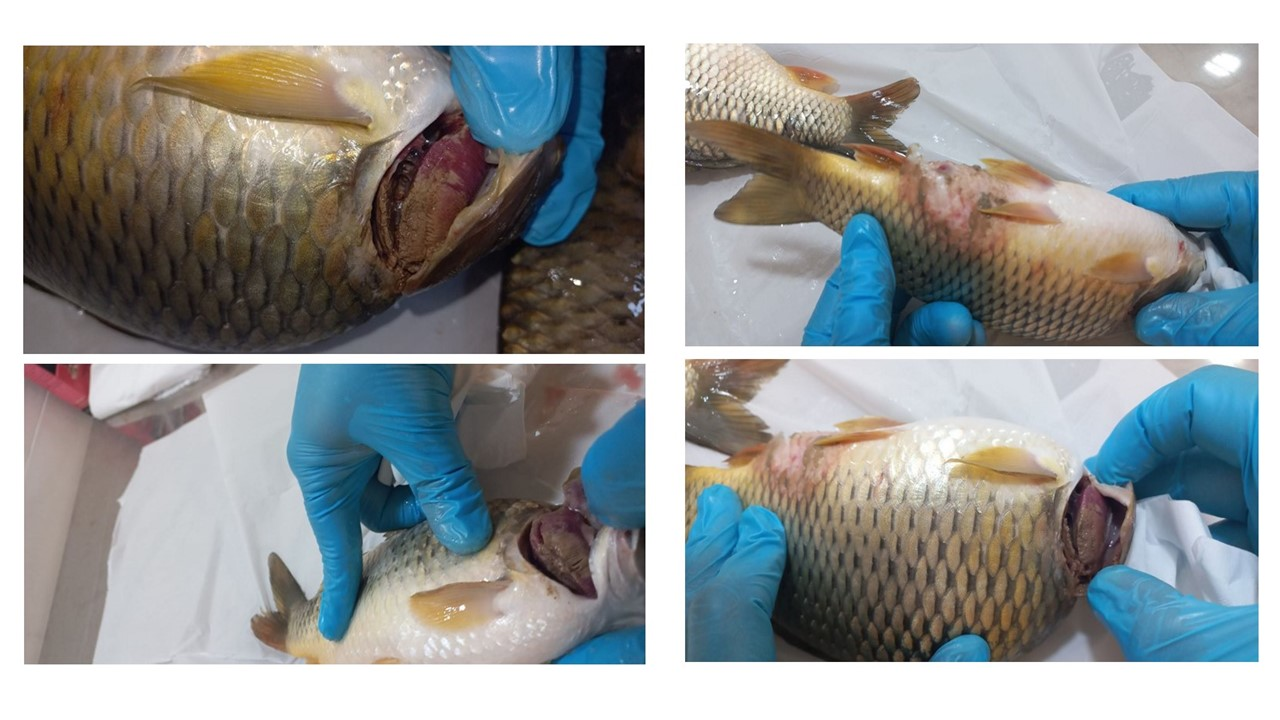

Supplement: S1 Fig — (TIF) [file pone.0303475.s001.tif]

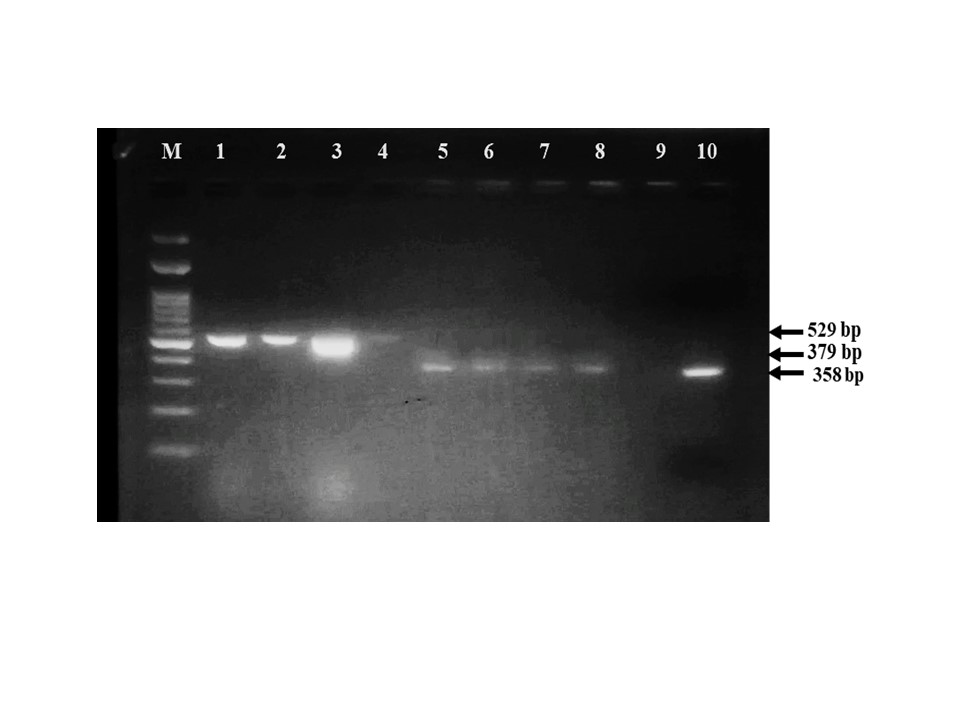

Supplement: S2 Fig — (TIF) [file pone.0303475.s002.tif]

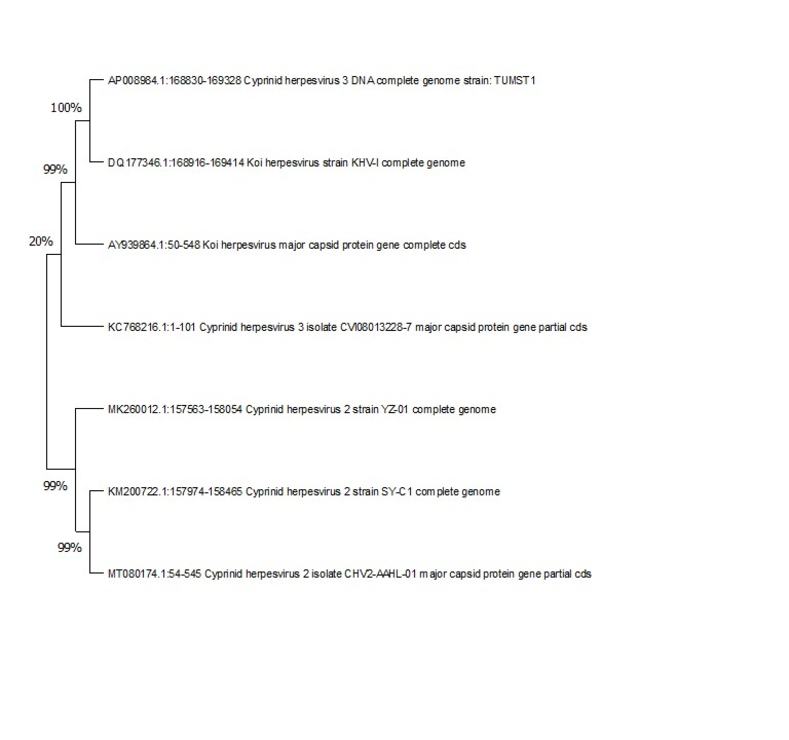

Supplement: S3 Fig — (TIF) [file pone.0303475.s003.tif]

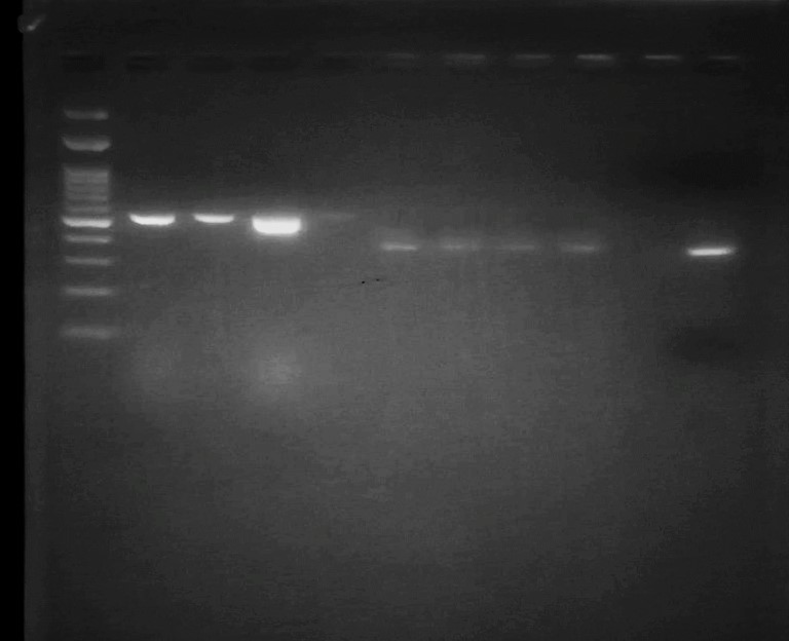

Supplement: S4 Fig — (TIF) [file pone.0303475.s004.tif]
