# Supplementary material for: First detection of Koi herpesvirus disease (KHVD) in Garmian, Kurdistan region of Iraq: A clinical and molecular study
Source: PLoS One. 2024 May 31;19(5):e0303475. doi: 10.1371/journal.pone.0303475 (PMC11142564; doi:10.1371/journal.pone.0303475)
Supplement: S1 File — (DOCX) [file pone.0303475.s005.docx]

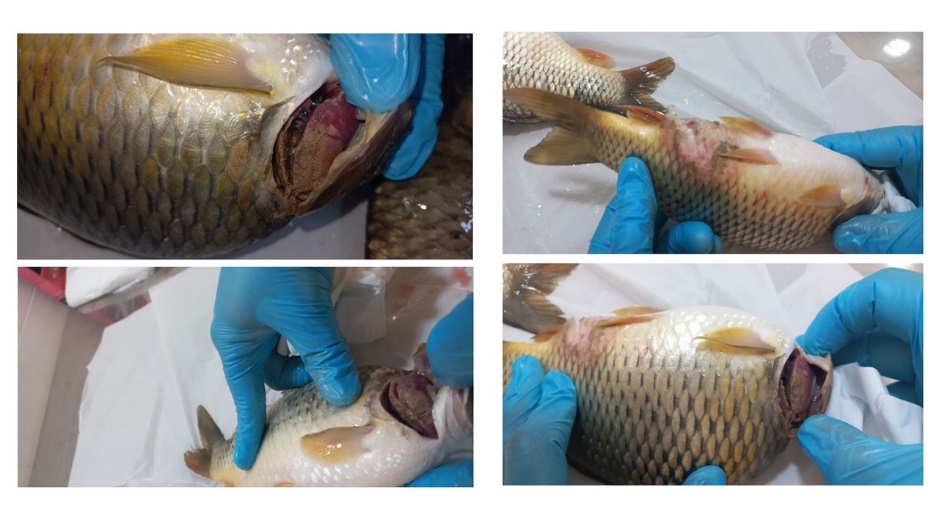


**Figure 1: Fish, carp, whole fish. Multiple variably sized, irregular skin ulcers gill discoloration and necrosis.**


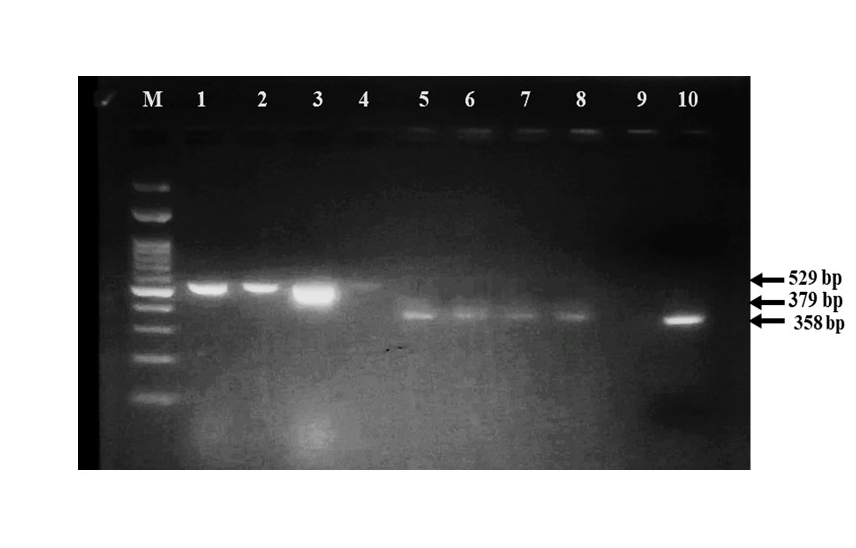


**Figure 2: Agarose gel electrophoresis Cyprinid herpesvirus-3 (CyHV-3) PCR and nested PCR product utilizing specific external and internal primer sets of the CyHV-3 major capsid protein gene (529 and 379 bp respectively) for samples from four fish farms. Separation is on a 1.5% agarose gel stained with ethidium bromide. Lanes are M: 100-bp DNA ladder, lanes1-4: Expected amplicon size of 529 bp in the four samples specific for major capsid protein gene of CyHV-3, Lane 5-8: show expected nested PCR amplicon size of 379 bp in the four samples, Lane 9: negative PCR control, Lane: Positive control, PCR amplifications of vertebrate cytB sequences (358 bp).**


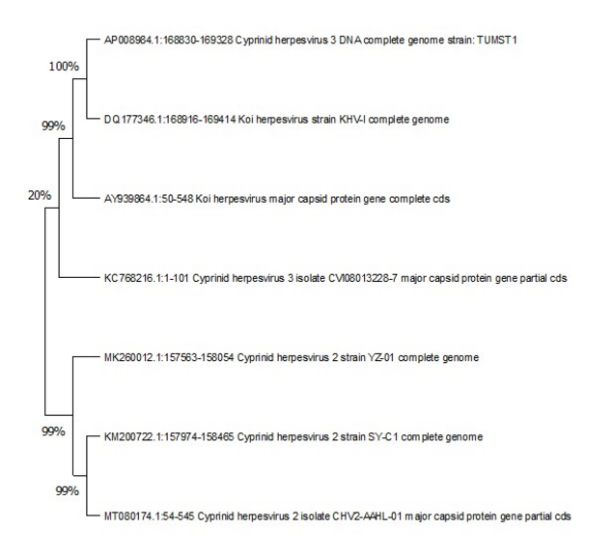


**Figure 3: Phylogenetic analysis of Kurdistan region isolates of koi herpesvirus (KHV) was conducted, comparing them with seven foreign CyHV-3 isolates based on the partial nucleotide sequences of the MCP gene. Initially, the ClustalW tool was employed to align nucleotide sequences, followed by construction of the Phylogenetic tree using the neighbor-joining method through the MEGA 11 program.**
