# Supplementary material for: First detection of Koi herpesvirus disease (KHVD) in Garmian, Kurdistan region of Iraq: A clinical and molecular study
Source: PLoS One. 2024 May 31;19(5):e0303475. doi: 10.1371/journal.pone.0303475 (PMC11142564; doi:10.1371/journal.pone.0303475)

PLOS ONE

First Detection of Koi herpesvirus Disease (KHVD) in Garmian, Kurdistan Region of Iraq: a clinical and molecular study

--Manuscript Draft--

**Manuscript Number:**	PONE-D-24-08826
**Article Type:**	Research Article
**Full Title:**	First Detection of Koi herpesvirus Disease (KHVD) in Garmian, Kurdistan Region of Iraq: a clinical and molecular study
**Short Title:**	First Detection of Koi herpesvirus Disease (KHVD) in Garmian, Kurdistan Region of Iraq
**Corresponding Author:**	Diyar Akbar<br>Tikrit University<br>Tikrit, IRAQ
**Keywords:**	Koi herpesvirus Disease, molecular, Kalar, Kurdistan Region, Iraq
**Abstract:**	Koi herpesvirus disease (KHVD) is attributed to cyprinid herpesvirus-3 (CyHV-3) and predominantly affects common carp and ornamental koi carp (Cyprinus carpio). This viral infection leads to substantial morbidity and mortality among these fish species. This study aimed to confirm the presence of KHVD in the Kurdistan region of Iraq by employing clinical and optimized molecular assays on fish populations experiencing high mortality among common carp in carp farm.<br><br>Methodology:<br>The present research was conducted in the Kalar district, situated at the heart of Garmian province in Iraqi Kurdistan. four samples from common carp fish farms were received by our laboratory. These samples specifically displaying clinical signs associated with koi herpesvirus (KHV) infection, were subjected to clinical examinations, and PCR assay in addition to sequence analysis.<br>Results:<br>The results of the current study revealed that the observed clinical signs, particularly gill necrosis, skin lesions, and sunken eyes, closely resembled the clinical signs of KHVD in common carp fish. In addition, PCR, nested PCR, and sequence analysis assay detected appropriate DNA fragments of the CyHV-3 major capsid protein gene confirming the first detection of KHVD in common carp fish in the Kurdistan region of Iraq.<br>Conclusion:<br>In this study, the results confirm the detection of KHVD in the Kurdistan region, Iraq, for the first time. This study revealed that CyHV-3 was responsible for KHVD-related signs and symptoms. Based on these results, it is strongly recommended that comprehensive studies be initiated to investigate the prevalence and distribution of CyHV-3.
**Order of Authors:**	Diyar Akbar
	Shaymaa Abdulaziz Nawokas Abdulaziz Nawokas
	Muhammad majed mardoukhi
**Additional Information:**	
**Question**	**Response**
**Financial Disclosure**<br><br>Enter a financial disclosure statement that describes the sources of funding for the work included in this submission. Review the submission guidelines for detailed requirements. View published research	The author(s) received no specific funding for this work.

Powered by Editorial Manager® and ProduXion Manager® from Aries Systems Corporation

articles from *PLOS ONE* for specific examples.<br><br>This statement is required for submission and **will appear in the published article** if the submission is accepted. Please make sure it is accurate.<br><br>**Funded studies**<br>Enter a statement with the following details:<br>• Initials of the authors who received each award<br>• Grant numbers awarded to each author<br>• The full name of each funder<br>• URL of each funder website<br>• Did the sponsors or funders play any role in the study design, data collection and analysis, decision to publish, or preparation of the manuscript?<br><br>Did you receive funding for this work?	
**Competing Interests**<br><br>Use the instructions below to enter a competing interest statement for this submission. On behalf of all authors, disclose any competing interests that could be perceived to bias this work—acknowledging all financial support and any other relevant financial or non-financial competing interests.<br><br>This statement is required for submission and **will appear in the published article** if the submission is accepted. Please make sure it is accurate and that any funding sources listed in your Funding Information later in the submission form are also declared in your Financial Disclosure statement.<br><br>View published research articles from *PLOS ONE* for specific examples.	The authors have declared that no competing interests exist

**NO authors have competing interests**<br><br>Enter: *The authors have declared that no competing interests exist.*<br><br>**Authors with competing interests**<br><br>Enter competing interest details beginning with this statement:<br><br>*I have read the journal's policy and the authors of this manuscript have the following competing interests: [insert competing interests here]*<br><br>* typeset	
**Ethics Statement**<br><br>Enter an ethics statement for this submission. This statement is required if the study involved:<br><br>• Human participants<br>• Human specimens or tissue<br>• Vertebrate animals or cephalopods<br>• Vertebrate embryos or tissues<br>• Field research<br><br>Write "N/A" if the submission does not require an ethics statement.<br><br>General guidance is provided below. Consult the submission guidelines for detailed instructions. **Make sure that all information entered here is included in the Methods section of the manuscript.**	N/A

**Format for specific study types**<br><br>**Human Subject Research (involving human participants and/or tissue)**<br>• Give the name of the institutional review board or ethics committee that approved the study<br>• Include the approval number and/or a statement indicating approval of this research<br>• Indicate the form of consent obtained (written/oral) or the reason that consent was not obtained (e.g. the data were analyzed anonymously)<br><br>**Animal Research (involving vertebrate animals, embryos or tissues)**<br>• Provide the name of the Institutional Animal Care and Use Committee (IACUC) or other relevant ethics board that reviewed the study protocol, and indicate whether they approved this research or granted a formal waiver of ethical approval<br>• Include an approval number if one was obtained<br>• If the study involved *non-human primates*, add *additional details* about animal welfare and steps taken to ameliorate suffering<br>• If anesthesia, euthanasia, or any kind of animal sacrifice is part of the study, include briefly which substances and/or methods were applied<br><br>**Field Research**<br><br>Include the following details if this study involves the collection of plant, animal, or other materials from a natural setting:<br>• Field permit number<br>• Name of the institution or relevant body that granted permission	
**Data Availability**<br><br>Authors are required to make all data underlying the findings described fully available, without restriction, and from the time of publication. PLOS allows rare exceptions to address legal and ethical concerns. See the PLOS Data Policy and FAQ for detailed information.	Yes - all data are fully available without restriction

A Data Availability Statement describing where the data can be found is required at submission. Your answers to this question constitute the Data Availability Statement and **will be published in the article**, if accepted.<br><br>**Important:** Stating 'data available on request from the author' is not sufficient. If your data are only available upon request, select 'No' for the first question and explain your exceptional situation in the text box.<br><br>Do the authors confirm that all data underlying the findings described in their manuscript are fully available without restriction?	
**Describe where the data may be found in full sentences. If you are copying our sample text, replace any instances of XXX with the appropriate details.**<br><br>• If the data are **held or will be held in a public repository**, include URLs, accession numbers or DOIs. If this information will only be available after acceptance, indicate this by ticking the box below. For example: *All XXX files are available from the XXX database (accession number(s) XXX, XXX.*).<br>• If the data are all contained **within the manuscript and/or Supporting Information files**, enter the following: *All relevant data are within the manuscript and its Supporting Information files.*<br>• If neither of these applies but you are able to provide **details of access elsewhere**, with or without limitations, please do so. For example:<br><br>*Data cannot be shared publicly because of [XXX]. Data are available from the XXX Institutional Data Access / Ethics Committee (contact via XXX) for researchers who meet the criteria for access to confidential data.*<br><br>*The data underlying the results presented in the study are available from (include the name of the third party*	All relevant data are within the manuscript and its Supporting Information files.

and contact information or URL).<br>• This text is appropriate if the data are owned by a third party and authors do not have permission to share the data.<br><br>* typeset	
Additional data availability information:	

Manuscript
Click here to access/download;Manuscript;Koi_pape_final_okok.docx

First Detection of Koi herpesvirus Disease (KHVD) in Garmian, Kurdistan Region of Iraq: a clinical and molecular study

Diyar Akbar Hasan **Al-Jaf** [1*], Shaymaa Abdulaziz **Nawokas** [2], Muhammad majed **mardoukhi** [3]

[1] College of Medicine, University of Garmian, Kalar, Iraq

[2] Biology Department, College of Education, University of Garmian, Kalar, Iraq

[3] Kalar Private Veterinary Clinic, Kalar, Iraq

*** Corresponding author:** Diyar Akbar Hasan Al-Jaf [1]; diyar.akbar@garmian.edu.krd; ORCID

Couthers: Shaymaa Abdulaziz Nawokas [2]**;** shaymaa.abdulaziz@garmian.edu.krd
Muhammad majed mardoukhi [3] Majedmardokhi@yahoo.com

abstract

Koi herpesvirus disease (KHVD) is attributed to cyprinid herpesvirus-3 (CyHV-3) and predominantly affects common carp and ornamental koi carp (Cyprinus carpio). This viral infection leads to substantial morbidity and mortality among these fish species. This study aimed to confirm the presence of KHVD in the Kurdistan region of Iraq by employing clinical and optimized molecular assays on fish populations experiencing high mortality among common carp in carp farm.

Methodology:

The present research was conducted in the Kalar district, situated at the heart of Garmian province in Iraqi Kurdistan. four samples from common carp fish farms were received by our laboratory. These samples specifically displaying clinical signs associated with koi herpesvirus (KHV) infection, were subjected to clinical examinations, and PCR assay in addition to sequence analysis.

Results:

The results of the current study revealed that the observed clinical signs, particularly gill necrosis, skin lesions, and sunken eyes, closely resembled the clinical signs of KHVD in common carp fish. In addition, PCR, nested PCR, and sequence analysis assay detected appropriate DNA fragments of the CyHV-3 major capsid protein gene confirming the first detection of KHVD in common carp fish in the Kurdistan region of Iraq.

Conclusion:

In this study, the results confirm the detection of KHVD in the Kurdistan region, Iraq, for the first time. This study revealed that CyHV-3 was responsible for KHVD-related signs and symptoms. Based on these results, it is strongly recommended that comprehensive studies be initiated to investigate the prevalence and distribution of CyHV-3.

KEYWORDS

Koi herpesvirus Disease, molecular, Kalar, Kurdistan Region, Iraq

1 .Introduction

Koi herpesvirus disease (KHVD) is a disease caused by cyprinid herpesvirus-3 (CyHV-3), primarily affecting common carp and ornamental koi carp (Cyprinus carpio) in Europe and Asia. It

results in significant morbidity and mortality in these fish and is characterized by clinical signs such as white patches, skin hemorrhages, lethargy, reduced appetite, sunken eyes, enlargement of the spleen and kidney, and gill necrosis[1].

CyHV3, belonging to the Alloherpesviridae family and the genus Cyprinivirus, possesses the lengthiest genome within the Herpesvirales order, consisting of a double-stranded DNA approximately 295 kbp in size [2, 3]. Using the genes responsible for viral DNA polymerase and the ATPase subunit of terminase as a reference, CyHV-3 is presently categorized alongside CyHV-1 and CyHV-2 in the Cyprinivirus genus within the Alloherpesviridae family. The mature viral particles are 170 to 200 nm in diameter. CyHV-3 can be detected by multiple PCR-based methods and by enzyme-linked immunosorbent assay [4, 5[illegible]

KHVD has decimated major carp populations in various countries such as Israel, Indonesia, Taiwan, Japan, Germany, Canada, and the United States. It has been a notifiable disease in Germany since 2005 and by the World Organization for Animal Health since 2007 and has spread to most regions of the world [6, 7].

In 1998, initial large-scale deaths of both common and koi carp were documented in Israel and the United States [8]. However, examinations of archived samples revealed that the virus had been in wild common carp since 1996 in the United Kingdom [9]. Shortly after the initial report, outbreaks of Cyprinid herpesvirus-3 (CyHV-3) were observed in various countries across Europe, Asia, and Africa. Currently, CyHV-3 has been identified worldwide, except in South America, Australia, and northern Africa [6]. Globally, CyHV-3 has led to substantial financial and economic losses in the koi and common carp aquaculture industries. In the countries neighboring Iraq, the first detection of koi herpesvirus (KHV) in koi occurred in Iran in 2015, as documented through clinical, histopathological, and molecular studies [10].

In late October 2018, Iraq reported the first documented case of a Koi herpesvirus (KHV) outbreak. The incident impacted the carp farming industry in the central Euphrates region of Iraq, resulting in the deaths of several fish. The virus responsible for this outbreak was identified as Cyprinid herpesvirus 3 (CyHV-3), the causative agent of KHVD. This marked the first known occurrence of KHV in Iraq and had a substantial effect on the country's carp farming sector [11].

Aquaculture practices in Iraq emerged in the mid-to-late 20th century, with the introduction of the common carp (Cyprinus carpio) in 1955 at the Al-Zaafaraniya fish farm in Baghdad ([12]. It is thriving in Iraq, primarily relying on the Tigris and Euphrates rivers for farming because of the country's limited coastline along the Gulf [13]. These freshwater fish farms exist in both the public

and private sectors, with their concentration mainly in the central and southern regions of Iraq. The production of freshwater fish in Iraq is predominantly centered on common carp (Cyprinus carpio carpio), although there is also limited cultivation of grass carp (Ctenopharyngodon idella) and silver carp (Hypophthalmichthys molitrix). In 2015, the total freshwater carp production in Iraq was estimated to be approximately 30,000 tons [14, 15].

The common carp industry in the Kurdistan region of Iraq developed only during the last twenty years or so, and now spread to many farms in the region[16] and it is a flourishing sector that contributes to economic growth. Common carp, a popular food fish, is cultivated as a cost-effective means of protein production. The industry has experienced significant growth recently, driven by an increasing demand for fish and governmental support. Numerous fish farms in rural areas near water sources, such as rivers and lakes, focus on raising common carp in ponds or tanks. These fish are fed a combination of commercial pellets and natural foods. Common carp's resilience, ability to tolerate poor water quality, and ability to withstand high temperatures make them well suited for aquaculture in the Kurdistan Region, particularly in areas with limited water resources [17]. currently no documented evidence of KHVD in the Kurdistan Region of Iraq.

The study could address the gap in understanding the prevalence and distribution of this virus in the region. The study aimed to confirm the presence of Koi herpesvirus Disease (KHVD) in the Kurdistan Region by employing clinical and molecular assays on fish populations experiencing high mortality among common carp in carp farms Iraqi Kurdistan region, with confirmation through sequence analysis

2. Materials and Methods

2.1. Study area

The present research was conducted in the Kalar district, situated at the heart of Garmian province in the Kurdistan region of Iraqi, positioned around 140 km southeast of Sulaymaniyah and 30 km from the Iranian border, the district encompasses a geographical area with a population of approximately 250,000 residents.

2.2. Sample collection

Our laboratory received samples from four farms of common carp fish located in Kalar district: two samples in December 2022, one sample in June 2023, and another sample in October 2023. Fish exhibiting clinical signs of koi herpesvirus (KHV) infection, including lethargy, anorexia, increased mucus secretion, skin lesions, gill discoloration and necrosis, and sunken eyes, were targeted for sampling.

2.3. DNA extraction

The extraction of genomic DNA from fish tissues was conducted in the following manner: the skin, gills, liver, kidney, heart, spleen, and brain tissues were finely sliced, followed by grinding and homogenization using a mortar and pestle with the addition of liquid nitrogen. The extraction of genomic viral nucleic acid from these tissues was accomplished using the AddPrep Viral Nucleic Acid Extraction Kit (Add Bio Inc, Korea) in accordance with the manufacturer's guidelines.

2.4. Primer design

Two sets of primers were designed for the specific detection of CyHV-3 in tissues of suspected samples. Previously, the primers were checked for homology and found to be specific to CyHV-3. The external primer pair amplified a 529 bp fragment of CyHV-3 DNA, and the internal primer pair amplified a 379 bp fragment of CyHV-3 DNA. These primers were previously described by El-Matbouli *et al.* (2007; GenBank Accession number AY939864) [18]. The external primer pair (sense: 5’–ACCAACTTTAGCACGGACGAC–3’; antisense: 5’–ACTTGACCAGGTACAGCAGC–3’) and the internal primer pair for nested PCR (sense: 5’–CTTTAGCACGGACGACTTTGGC–3’; antisense: 5’–GTAGACGATGGACAGGGAG–3’). We used the primers introduced by Kocher et al. (1989) and Boakye et al. (1999) for amplification of a portion (358 bp) of CytB gene of vertebrate mtDNA as positive control [19, 20].

2.5. PCR.

PCR amplification was performed using 20 µl total volume as follows: the reactions mixture was prepared by mixing 10 µl Add start mastermix 2 x (Add Bio Inc, Korea) with 1 µl of each external primer (5 pM) and 5 µl genomic DNA in addition to 3 µl of Nuclease-Free Water. The reactions were run in a thermal cycler (Applied Biosystems 2720, Foster City, CA 94404 USA) by initial denaturation at 95°C for 5 minutes; followed by 40 cycles of 95°C for 30 seconds, annealing temperature at 60°C for 45 seconds and 72°C for 30 seconds; and finally extended at 72°C for 5 minutes. The specific PCR amplification protocol used for nested PCR was the same as the protocol used for the first PCR but with the internal set of primer. The only difference was that the genomic DNA template for the nested PCR was the amplification product of the first PCR [18].

The amplified PCR products obtained from the first PCR and the nested PCR were separated using gel electrophoresis on a 1.5% agarose gel. The electrophoresis buffer used was Tris-Borate-EDTA-Na_2 (TBE) buffer. The amplified PCR products were visualized by staining the gel with ethidium bromide[illegible]o determine the molecular size of the PCR and nested PCR amplicons, a 100 bp DNA ladder from GeneDireX, Inc, was used as a reference.

2.7. Ethical approval

The ethical committee at the Research Centre, University of Garmian granted the necessary ethical approval for the study.

3. Results

3.1. clinical findings

Upon clinical examination, changes in gill appearance were the most prominent clinical finding. The gills of the affected fish exhibited gross changes, including varying degrees of discoloration and necrosis, accompanied by an excessive production of slimy mucus The fish's eyes were noticeably sunken. Skin ulcerations were also among prominent clinical finding, with lesions varying in size and scattered throughout the body. These ulcers merged and could reach up to 2cm in diameter (Figure 1).

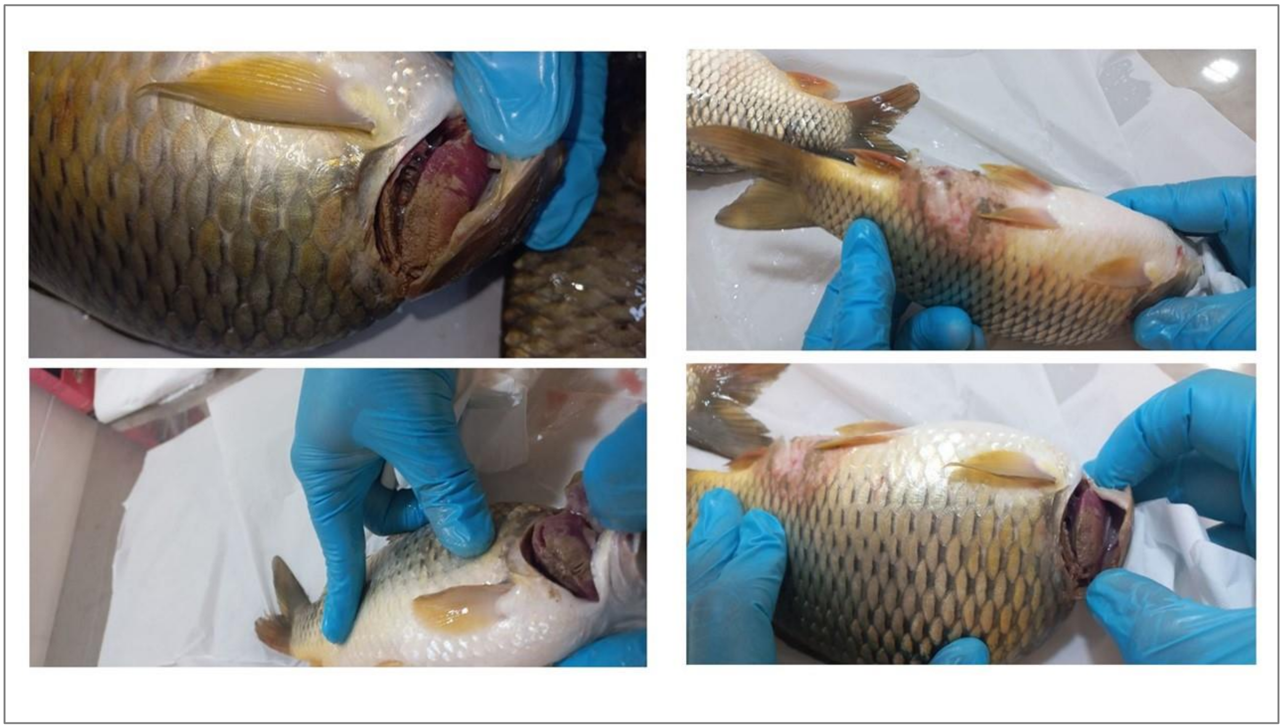

Figure 1: Fish, carp, whole fish. Multiple variably sized, irregular skin ulcers gill discoloration and necrosis

3.2. PCR detection

Positive results were detected when fragments of the appropriate size of 529 bp for PCR and 379 bp for nested PCR of the CyHV-3 major capsid protein (MCP) gene (accession number AY939864) [21] was amplified from the CyHV-3 suspected samples (Figure 2).

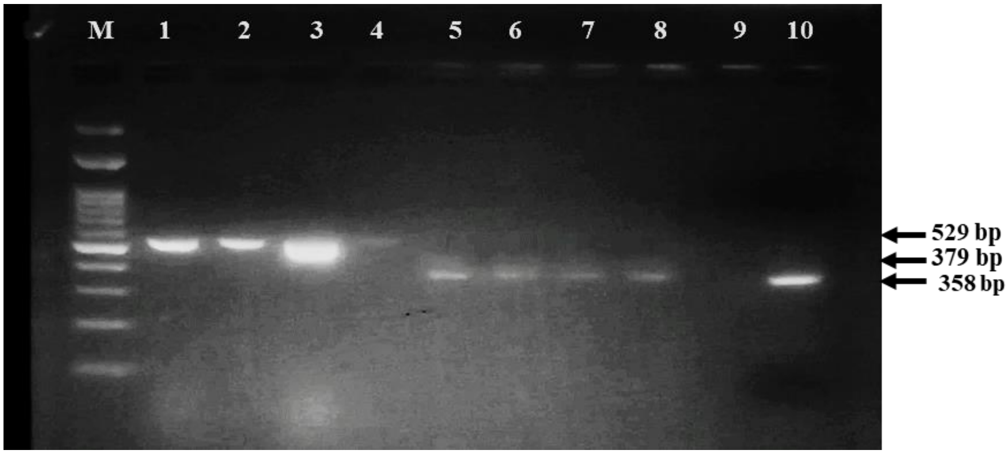

Figure 2: Agarose gel electrophoresis Cyprinid herpesvirus-3 (CyHV-3) PCR and nested PCR product utilizing specific external and internal primer sets of the CyHV-3 major capsid protein gene (529 and 379 bp respectively) for sample from four fish farms. Separation is on a 1.5% agarose gel stained with ethidium bromide. Lanes are M: 100-bp DNA ladder, lanes1-4: Expected amlicon size of 529 bp in

the four samples specific for major capsid protein gene of CyHV-3, Lane 5-8: show expected nested PCR amplicon size of 379 bp in the four samples, Lane 9: negative PCR control, Lane: Positive control, PCR amplifications of vertebrate cytB sequences (358 bp).

Sequence analysis

Partial sequence analysis of the major capsid protein (MCP) gene, were identical with that of several KHV strains in the GenBank database: examples TUMST1 (Japan, accession number AP008984) and KHV-I (Israel, DQ177346).

4. Discussion

The study aimed to verify the existence of Koi herpesvirus Disease (KHVD) in the Kurdistan Region by utilizing clinical and molecular assays including sequence analysis on fish populations experiencing mass mortality among common carp in carp farms within the Iraqi Kurdistan region, with confirmation through sequence analysis. We describe the first detection of KHV in common carp fish in Iraqi Kurdistan region because no study addressed KHVD before this region , but in Iraq, KHVD outbreak that started in late October 2018 [22]. The Disease occurred when the water temperature was optimal for the growth and replication of CyHV-3, which is between 18-28 °C but no mortality was observed for CyHV-3-exposed fish at 13°C [23]. KHVD was reported by four countries during the period from 1 January 2018 to 20 March 2019. The disease was first reported in Romania in June 2018, and then in Canada in September 2018, Italy in December 2018, and Iraq in January 2019. As of 20 March 2019, there are still ongoing KHVD outbreaks in Romania, Italy, and Iraq.[24]

Clinical signs of KHVD in fish include pale and necrotic gills, enophthalmus, and excessive secretion of mucus[5]. Similar clinical signs were observed in the present study, especially gill necrosis, skin lesions and sunken eye.

For rapid and accurate diagnosis of acute KHV infections, PCR-based techniques have been shown to be the most sensitive diagnostic tools for KHV detection, as reported by Haenen et al. [25, 26]. Our laboratory used the CyHV-3 major capsid protein gene-based PCR protocol with external and internal primers to amplify 529 and 379 bp fragments from CyHV-3 DNA. The sensitivity of this PCR protocol has been well addressed before by El-Matbouli et al. [18].

The results confirmed that KHV behand the KHVD related clinical signs in four fish samples tested by mentioned PCR protocol. previously no microbiological and molecular study conducted on viral disease in common carp fish farms in the region, while most studies concentrated on bacterial and protozoal disease[27-29]. Outbreaks of the disease in this region have occurred in

different period of the years without regard to temperature, may be due to the fact the disease new to region and it is first exposure to CyHV-3. The presence of secondary parasitic infections along with CyHV-3 in diseased carp can complicate the pathogenesis of the disease [22]. These secondary infections are prevalent in CyHV-3-affected carp and can worsen the clinical symptoms and increase mortality rates[30].

The swift and widespread of KHV is likely attributed to a variety of factors, such as the international trade in ornamental and aquarium fish, deficiencies in historical diagnostic methods, and the absence of regulations for controlling and preventing the virus's introduction[31].

The precise origin and mode of introduction of CyHV-3 to carp farms in the Kurdistan region of Iraq remain undetermined. However, based on existing research, several scenarios can be hypothesized. The most probable pathways are the movement of live fish between fish farms in this region and the rest of Iraq or the importation of carp. In addition, wild non-cyprinid fish could pose a potential risk factor for CyHV-3 infection [32].

Another reason that makes tracing the source of the disease challenging is the ability of KHV to establish latent infections. This means that the virus can maintain its DNA in fish hosts without causing any visible symptoms or signs of disease. Latent KHV infections have been reported, particularly in fish presumed to have been previously exposed to KHV [33]. The virus can reemerge from its dormant state when environmental factors such as temperature fluctuations create favorable conditions for its growth [34].

To prevent the spread of KHV, early detection is paramount. PCR, a powerful technique capable of detecting minute viral quantities, is deemed effective for identifying KHV latency in common carp [35]. Therefore, this study employed previously optimized PCR assay [18] to assess the presence of KHV, particularly in common carp from Kalar district, Kurdistan.

5. Conclusions:

In this study, the results confirm the detection of KHVD in Kurdistan, Iraq, for the first time in common carp (*Cyprinus carpio*) freshwater farms by Research study. Clinical and molecular examination revealed that CyHV-3 was responsible for Koi herpesvirus disease (KHVD) related signs, symptoms, and mortality.

6. Recommendation:

Based on this result, it is strongly recommended to initiate comprehensive studies aimed at assessing the prevalence and distribution of CyHV-3 in diverse carp farms across the Kurdistan

region. Furthermore, conducting genetic analyses of CyHV-3 isolates in Kurdistan will contribute to the development of efficient strategies for managing and preventing the spread of the disease in the region.

Conflict of interest: All authors have no conflict of interest to declare.

Data availability statement: The data of this study are available upon request, from the corresponding author: Diyar Akbar Hasan Al-Jaf; diyar.akbar@garmian.edu.krd

References

Uncategorized References

1. Abdulraheem, A.L., et al., *Most Reliable Haematological Indices for Diagnosis of Iron Deficiency Anaemia from Non-Iron Deficiency Anaemia in Reproductive-Age Females.* 2023. **5**(1): p. 24-29.

2. Aoki, T., et al., *Genome sequences of three koi herpesvirus isolates representing the expanding distribution of an emerging disease threatening koi and common carp worldwide.* 2007. **81**(10): p. 5058-5065.
3. Hutoran, M., et al., *Description of an as yet unclassified DNA virus from diseased Cyprinus carpio species.* 2005. **79**(4): p. 1983-1991.
4. Michel, B., et al., *Cyprinid herpesvirus 3.* 2010. **16**(12): p. 1835.
5. Gotesman, M., et al., *CyHV-3: the third cyprinid herpesvirus.* 2013. **105**(2): p. 163-174.
6. Pokorova, D., et al., *Current knowledge on koi herpesvirus (KHV): a review.* 2005. **50**(4): p. 139-148.
7. Fisheries, F., *The State of World Fisheries and Aquaculture-2010 (SOFIA).* 2010.
8. Hedrick, R., et al., *A herpesvirus associated with mass mortality of juvenile and adult koi, a strain of common carp.* 2000. **12**(1): p. 44-57.
9. Walster, C.J.F.V.J., *Clinical observations of severe mortalities in koi carp, Cyprinus carpio, with gill disease.* 1999. **3**(3): p. 54-8.
10. Rahmati- Holasoo, H., et al., *First detection of koi herpesvirus from koi, Cyprinus carpio L. experiencing mass mortalities in Iran: clinical, histopathological and molecular study.* 2016. **39**(10): p. 1153-1163.
11. Ababneh, M., W. Hananeh, and M.J.H. Alzghoul. *Mass mortality associated with koi herpesvirus in common carp in Iraq*. 2020 [cited 6 8].
12. Mhaisen, F.J.I.J.V.S., *A review on the parasites and diseases in fishes of ponds and farms of Iraq.* 1993. **6**(2): p. 20-28.
13. El-Sayed, A.-F.M.J.F.F. and A. Circular, *Regional review on status and trends in aquaculture development in the Near East and North Africa-2015.* 2017(C1135/6): p. I.
14. Sadiddin, A., et al., *Are Iraqi displaced farmers returning to agriculture?* 2023: Food & Agriculture Org.
15. Dyck, M., *Potential for Aquaculture Integration with Wastewater Treatment Wetlands in Southern Iraq.* 2017.
16. Coad, B.W.J., *Freshwater fishes of Iraq.* 2010.
17. Salman, N.A., K.I. Saleh, and M.M.J.B.J.o.A.S. Taher, *Cage Culture in Iraq, Current Status & Potential: A Review.* 2022. **35**(2): p. 362-372.
18. El-Matbouli, M., U. Rucker, and H.J.D.o.A.O. Soliman, *Detection of Cyprinid herpesvirus-3 (CyHV-3) DNA in infected fish tissues by nested polymerase chain reaction.* 2007. **78**(1): p. 23-28.
19. Boakye, D., et al., *Identification of bloodmeals in haematophagous Diptera by cytochrome B heteroduplex analysis.* 1999. **13**(3): p. 282-287.
20. TD, K.J.P.N.A.S.U., *Dynamics of mitochondrial DNA evolution in animals: Amplifications and sequencing with conserved primers.* 1989. **86**: p. 189-191.
21. Waltzek, T.B., et al., *Koi herpesvirus represents a third cyprinid herpesvirus (CyHV-3) in the family Herpesviridae.* 2005. **86**(6): p. 1659-1667.
22. Toffan, A., et al., *First detection of koi herpesvirus and carp oedema virus in Iraq associated with a mass mortality in common carp (Cyprinus carpio).* 2020. **67**(2): p. 523-528.
23. Gilad, O., et al., *Molecular comparison of isolates of an emerging fish pathogen, koi herpesvirus, and the effect of water temperature on mortality of experimentally infected koi.* 2003. **84**(10): p. 2661-2667.
24. Kuribreña, M.A., et al., *Current animal health situation worldwide: analysis of events and trends.* 2019: p. 34.

25. Bergmann, S.M., et al., *Investigation on the diagnostic sensitivity of molecular tools used for detection of koi herpesvirus.* 2010. **163**(2): p. 229-233.
26. Monaghan, S., et al., *Sensitivity of seven PCR s for early detection of koi herpesvirus in experimentally infected carp, C yprinus carpio L., by lethal and non‐ lethal sampling methods.* 2015. **38**(3): p. 303-319.
27. Obaid, H.M., et al., *Common Carp (Cyprinus carpio) parasites diversity and prevalence in Erbil aquacultures: gills, skin and intestinal infections.* 2021. **13**(1): p. 34-41.
28. Mama, K.S. and S.M.J.P.A.E.T. Abdullah, Norway, *Infections of common carp Cyprinus carpio with ciliated protozoans parasites from Ainkawa fish hatchery in Kurdistan region, Iraq.* 2013: p. 9-13.
29. Mala, H.H., S.M.J.Z.J.o.P. Abdullah, and A. Sciences, *Isolation and identification of some Bacterial Species from Common carp (Cyprinus carpio Linnaeus, 1758) in Taqtaq District in Erbil Province, Kurdistan Region, Iraq.* 2022. **34**(5): p. 131-140.
30. Haenen, O., et al., *The emergence of koi herpesvirus and its significance to European aquaculture.* 2004. **24**(6): p. 293-307.
31. Garver, K.A., et al., *Mass mortality associated with koi herpesvirus in wild common carp in Canada.* 2010. **46**(4): p. 1242-1251.
32. Fabian, M., A. Baumer, and D.J.J.o.F.D. Steinhagen, *Do wild fish species contribute to the transmission of koi herpesvirus to carp in hatchery ponds?* 2013. **36**(5): p. 505-514.
33. Xu, J.-R., et al., *Analysis of koi herpesvirus latency in wild common carp and ornamental koi in Oregon, USA.* 2013. **187**(2): p. 372-379.
34. St-Hilaire, S., et al., *Reactivation of koi herpesvirus infections in common carp Cyprinus carpio.* 2005. **67**(1-2): p. 15-23.
35. Meyer, K., et al., *Detection of Koi herpesvirus: impact of extraction method, primer set and DNA polymerase on the sensitivity of polymerase chain reaction examinations.* 2012. **43**(6): p. 835-842.

Click here to access/download;Figure;figure 1.jpg

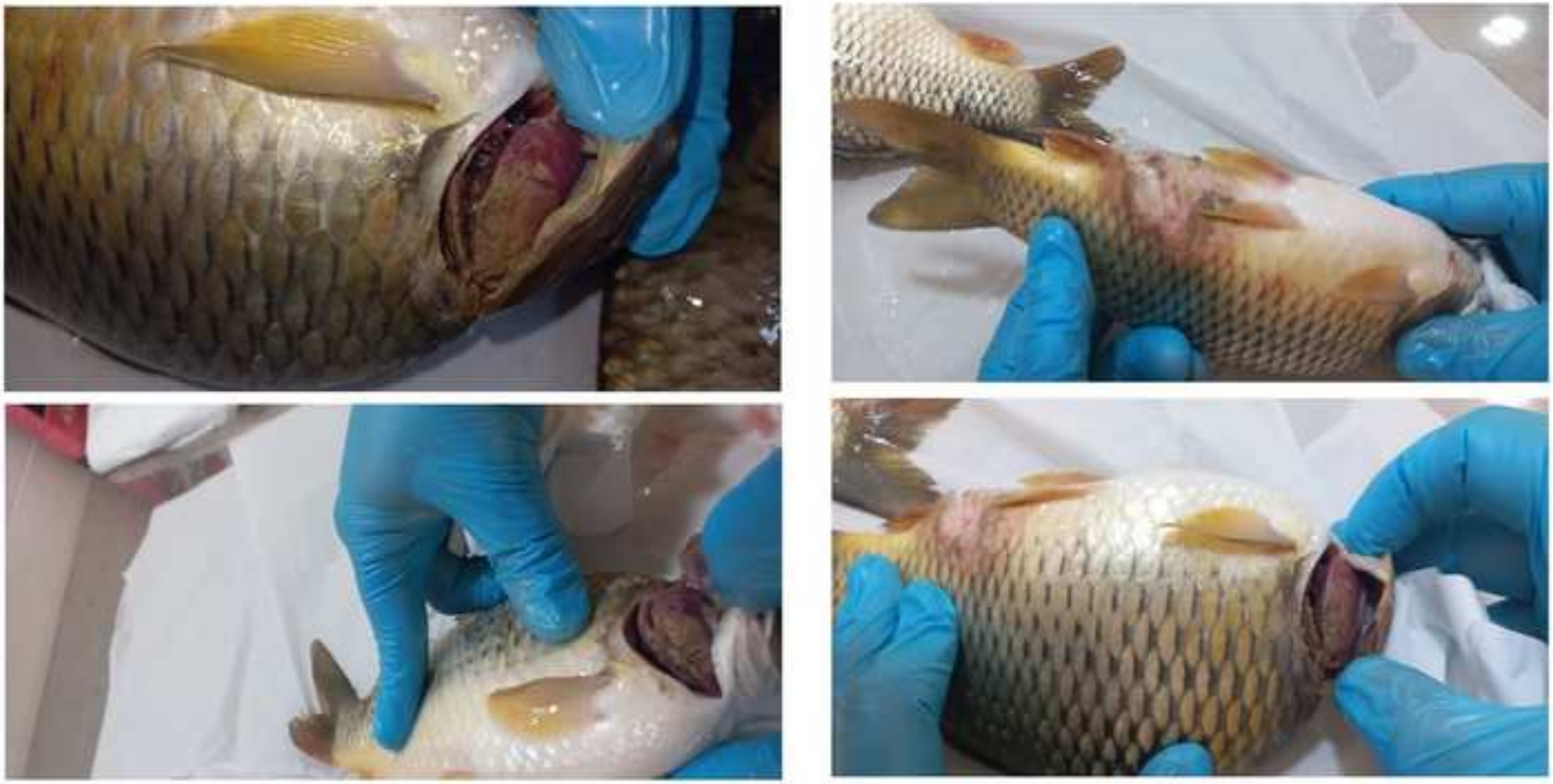

Click here to access/download;Figure;Figure 2.jpg

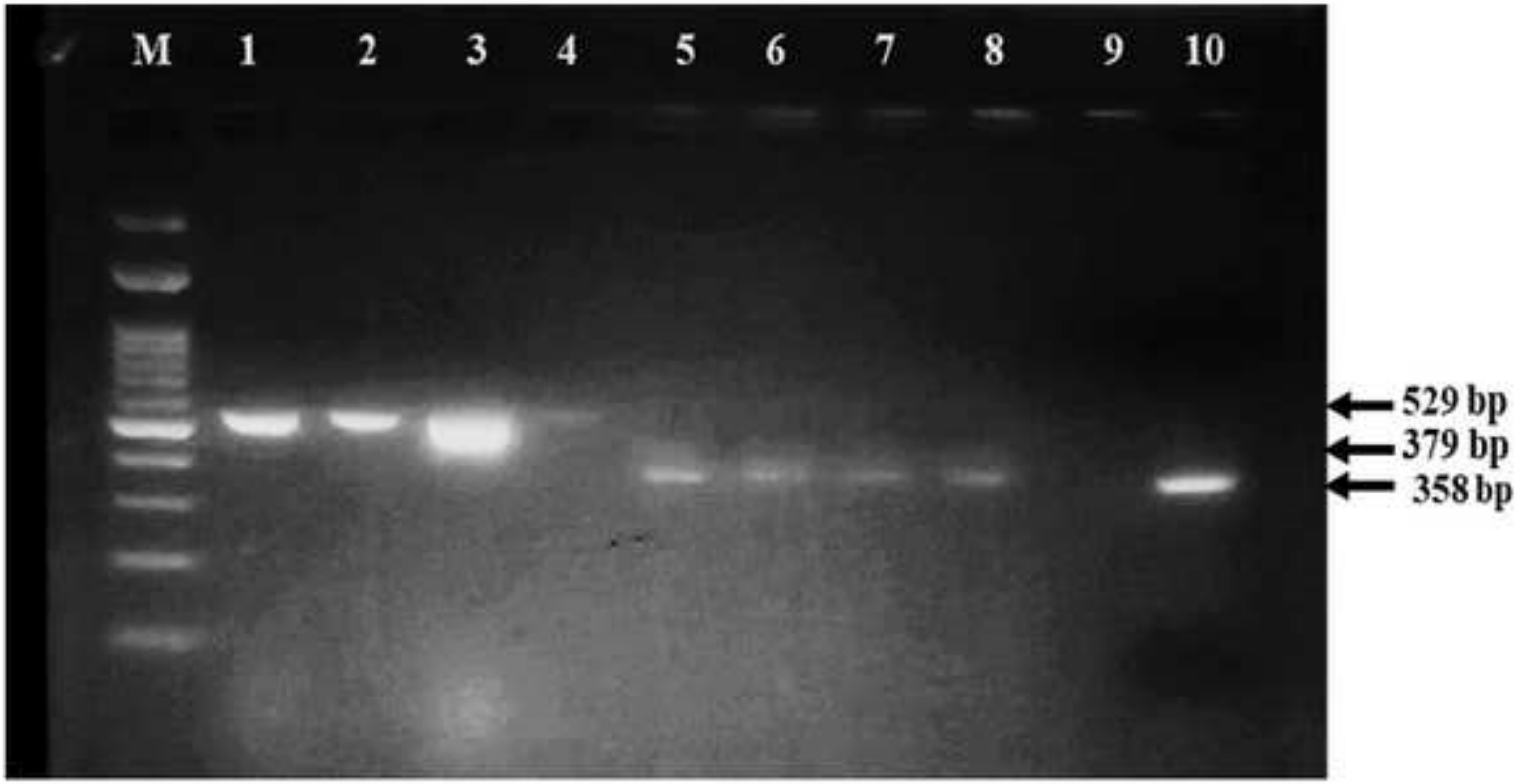

Supplement: Attachment — Submitted filename: PONE-D-24-Herpes virus-12-3-2024 after review.pdf [file pone.0303475.s006.pdf]
