# Supplementary material for: First detection of Koi herpesvirus disease (KHVD) in Garmian, Kurdistan region of Iraq: A clinical and molecular study
Source: PLoS One. 2024 May 31;19(5):e0303475. doi: 10.1371/journal.pone.0303475 (PMC11142564; doi:10.1371/journal.pone.0303475)
Supplement: Attachment — Submitted filename: Response to reviewers.pdf [file pone.0303475.s007.pdf]

Dear Editor and Reviewers

We would like to express our gratitude to you for your thoughtful evaluation of our manuscript titled " **First Detection of Koi herpesvirus Disease (KHVD) in Garmian, Kurdistan Region of Iraq: a clinical and molecular study**" We have carefully reviewed your comments and suggestions and have made the necessary revisions to enhance the quality and clarity of our research. Thank you for confirming the clarity and correctness of the language in our manuscript. Your review was priceless and greatly appreciated. Thank you.

1- Regarding the suggestion to include references on topics unrelated to our study. However, we respectfully assert that these references are not directly pertinent to the focus of our study, which centers on the examination of koi virus in common carp fish within the realm of aquaculture virology:

The suggest references by the Reviewers:

1) Al-Kanaan BM, Al-Ouqaili MT, Al-Rawi KF. Comparative study of the molecular, biochemical, and other parameters in Iraqi hepatitis B patients. Drug Invention Today. 2020 Jun 15;14(6).
2) Al-Ouqaili MT. Biofilm antimicrobial susceptibility pattern for selected antimicrobial agents against planktonic and sessile cells of clinical isolates of staphylococci using MICs, BICs and MBECs. Asian Journal of Pharmaceutics (AJP). 2018;12(04).
3) Bhat, R., Tandel, R. and Pandey, P.K., 2022. Alternatives to antibiotics for combating the antimicrobial resistance in aquaculture. Indian J Animal Health, 61, pp.01-18.
4) Bhat, R.A.H., Rehman, S., Tandel, R.S., Dash, P., Bhandari, A., Ganie, P.A., Shah, T.K., Pant, K., Yousuf, D.J., Bhat, I.A. and Chandra, S., 2021. Immunomodulatory and Antimicrobial potential of ethanolic extract of Himalayan Myrica esculanta in Oncorhynchus mykiss: Molecular modelling with Aeromonas hydrophila functional proteins. Aquaculture, 533, p.736213.
5) Bhat, R.A.H. and Altinok, I., 2023. Antimicrobial Resistance (AMR) and Alternative Strategies for Combating AMR in Aquaculture. Turkish Journal of Fisheries and Aquatic Sciences, 23(11).
6) Bhat, R.A.H., Khangembam, V.C., Pant, V., Tandel, R.S., Pandey, P.K. and Thakuria, D., 2024. Antibacterial activity of a short de novo designed peptide against fish bacterial pathogens. Amino Acids, 56(1), pp.1-13.

2- Since the fish samples were already deceased, note that no anesthesia or analgesia was administered to minimize suffering.

3- I have provided the original uncropped and unadjusted images underlying all blot or gel results as a supplementary file labeled or named as “**S4 fig original**” with the other figures, because I did know how to use the link related to original image data: https://journals.plos.org/plosone/s/figures#loc-original-images-for-blots-and-gels.

4- We confirm that all relevant data and its Supporting Information files are within the manuscript as confirmed by the reviewers by replying “Yes” to the question related to this subject.

5- I have changed reference number one (1) with the current reference because the former reference was mistakenly inserted from the Endnote library.

6- The viral Nucleic acid extraction procedure or protocol was the only one that was not included in the methodology because it is available online at the manufacturer’s website in a PDF file and can be downloaded

Sincerely,

Diyar Akbar Hasan al-Jaf
